# Identifying factors improving the intention to use antibiotics appropriately in children and adults using protection motivation theory

**DOI:** 10.1016/j.pecinn.2024.100293

**Published:** 2024-05-22

**Authors:** Hitomi Kawamura, Keiko Kishimoto

**Affiliations:** a Division of Social Pharmacy, Department of Healthcare and Regulatory Sciences, Graduate School of Pharmacy, Showa University, 1-5-8 Hatanodai, Shinagawa-ku, Tokyo 142-8555, Japan; b Department of Pharmacy, Tokyo Rosai Hospital, 4-13-21 Omoriminami, Ota-ku, Tokyo 143-0013, Japan

**Keywords:** Antibiotics, Children, Adults, Protection motivation theory, Awareness activities

## Abstract

**Objective:**

This study aimed to employ hypothetical models based on the protection motivation theory (PMT) to identify factors that improve the intention to use antibiotics appropriately (intention) among individuals who take antibiotics or administer them to their children.

**Methods:**

Adult Japanese participants, including 600 parents who administer antibiotics to children aged <14 years and 600 adults who take them, completed an online survey. Structural equation modeling (SEM) was conducted on hypothetical models representing intention using 19 questions based on PMT. If the hypothesized model did not fit, SEM was repeated to search for a new model.

**Results:**

The hypothesized models did not fit. Two factors were extracted from SEM: “understanding the risk of antimicrobial resistance” and “excessive expectation of antibiotics.” In adults, SEM revealed that “excessive expectation of antibiotics” (β = −0.50, *p* < 0.001) negatively influenced intention; in children, “excessive expectation of antibiotics” (β = −0.52, *p* < 0.001) negatively influenced intention, while “understanding the risk of antimicrobial resistance” (β = 0.22, *p* < 0.001) positively influenced intention.

**Conclusion:**

Factors influencing intention varied between adult and pediatric antibiotic use.

**Innovation:**

Awareness activities for appropriate antibiotic use should be tailored to population characteristics.

## Highlights

• This study evaluated factors influencing intention to use antibiotics appropriately.
• Factors differed between adult and pediatric antibiotic use.
• Excessive expectation of antibiotics affected pediatric and adult antibiotic use.
• In children, understanding the risk of antimicrobial resistance was also a factor.
• Awareness activities for proper antibiotic use differ for children and adults.

## Graphical abstract

**Figure:**
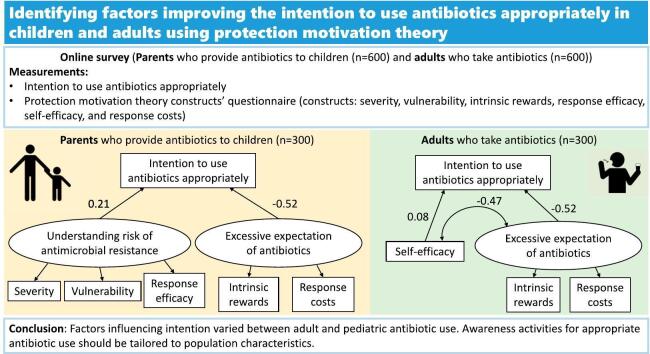

## 1 Introduction

Antimicrobial resistance is a threat to public health globally. The increase in antibiotic resistance has been hypothesized to be due to excessive antibiotic use [1]. The National Action Plan on Antimicrobial Resistance (2016–2020) [2] introduced by the Japanese government in 2016, includes programs for public awareness and education to prevent further increase in antimicrobial resistance. The AMR Clinical Reference Center provides measures against antimicrobial resistance for the general public [3]. Furthermore, awareness-raising activities have been conducted for mass media and the general public [[4], [5], [6]]. Nevertheless, the general public's understanding of antibiotics has changed minimally in recent years [7]. The percentage of the population with the correct knowledge about antibiotics is lower in Japan than in European countries [8]. Moreover, 63.1% of physicians who diagnose patients with the common cold without the need for antibiotic prescription reported that they would prescribe antibiotics if requested by the patients or their family members or when they are not convinced by the explanation provided [9]. Thus, the inappropriate use of antibiotics based on patients' requests needs to be reduced.

**Fig. 1:**
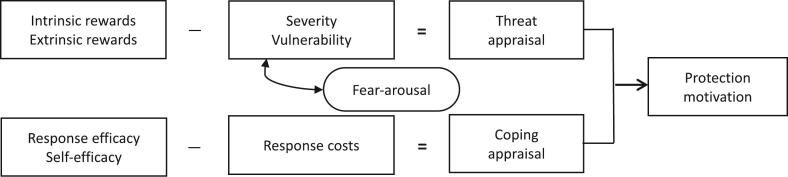
Protection motivation theory. Figure representing the protection motivation theory. It is assumed that “protection motivation” is formed by threat appraisal, consisting of severity and vulnerability, extrinsic and intrinsic rewards, and coping appraisal, consisting of response efficacy, self-efficacy, and response costs.

Approximately 90% of antibiotics used in Japan are oral antibiotics and are used more frequently in pediatric outpatients aged 0–14 years [10]. Hence, raising of awareness among Japanese parents is important. In a previous survey [11], we reported that Japanese mothers who were skeptical regarding antibiotics not being prescribed for their children lacked knowledge of antibiotics. Furthermore, excessive expectations based on personal experiences and concerns about children's illnesses resulted in dissatisfaction among Japanese mothers when antibiotics were not prescribed. Therefore, effective persuasion is necessary to convince parents that antibiotics are not always necessary. Protection motivation theory (PMT) [12], a model of persuasive communication, has been applied in the field of health behavior research [[13], [14], [15]]. This theory postulates that the motivation for protection is evoked by two processes: threat appraisal and coping appraisal (Fig. 1). No studies have evaluated the potential of PMT in predicting the intention to use antibiotics appropriately. The Appropriate Antibiotic Use Self-Efficacy Scale has been used to identify potential predictors of antibiotic use behaviors [16]. However, various factors are associated with patients' requests for antibiotics [11]. PMT evaluates the perceived seriousness of the issues and the burden associated with preventive behavior. Thus, PMT can potentially be used to evaluate the intention to appropriately use antibiotics across multiple dimensions. Protection motivation is often expressed as behavioral intentions [17]. In this study, we consider that protection motivation can be defined as the intention to use antibiotics appropriately. Excessive expectation of antibiotics for administration to children is associated with parental concerns. Thus, adults who take antibiotics and parents who administer them to their children may have different factors that influence their intention to use antibiotics appropriately. Thus, the main objective of this study was to evaluate the potential of PMT in assessing the intention to use antibiotics appropriately and identify factors that enhance the intention to use antibiotics appropriately, with the goal of educating the general public on the proper use of antibiotics. We also assessed differences in factors that influence the intention to appropriately use antibiotics among parents who administer antibiotics to their children and adults who take antibiotics.

## 2 Methods

This nationwide cross-sectional online survey was conducted between January 25 and 28, 2022. This study was approved by the Showa University Research Ethics Review Board (Ethical approval number: 21–130-A).

First, we constructed a hypothetical model to explain the intention to use antibiotics appropriately based on PMT. To evaluate this model, we conducted surveys about parents' intent to use antibiotics appropriately for pediatric antibiotic use (survey 1) and adults' intent to use antibiotics appropriately in their own antibiotics use (survey 2). An explanation of the study was provided on the survey screen and consent was obtained from all participants prior to initiation of the survey.

### 2.1 Measurements

The measurements comprised a PMT questionnaire, the intention to use antibiotics appropriately, and various characteristics. Because no PMT-based criteria have been established yet, we developed and refined the questionnaire items. PMT comprises seven constructs, which are the perception of the seriousness of the threat event (severity), probability that threat events may occur (vulnerability), physical pleasure and mental satisfaction from maladaptive behaviors (intrinsic rewards), social praise for maladaptive behaviors (extrinsic rewards), perception of the effectiveness of preventive behavior against the threat (response efficacy), assurance of one's ability to perform the preventive behavior (self-efficacy), and burden of performing the preventive behavior (response costs). The seven PMT constructs represent the perceptions of preventive behaviors and threat events. Some preventive behaviors did not necessarily fit in any of the seven constructs of PMT [18]. As the possibility of social acclaim due to the desire to prescribe unnecessary antibiotics is limited, we considered extrinsic rewards unnecessary and thus established six constructs: severity, vulnerability, intrinsic rewards, response efficacy, self-efficacy, and response costs.

Further, we considered whether variation in preventive actions or threat events would be more appropriate to represent each PMT construct with multiple items and to assess its validity. Therefore, we created pattern A with variations in preventive behaviors and pattern B with variations in threat events. Patterns A and B comprised 19 items each. Questionnaire items were developed based on previous literature [14,19,20]. We used a 7-point Likert scale ranging from 1 (strongly disagree) to 7 (strongly agree). Two online surveys, patterns A and B, were conducted as survey 1 (parents' intent to use antibiotics appropriately for pediatric antibiotic use), and two online surveys, patterns A and B, were conducted as survey 2 (adults' intent to use antibiotics appropriately in their own antibiotics use). In survey 1, participants were asked to answer all questions in a hypothetical situation where a child is administered antibiotics (Appendix A, Appendix B, Appendix C, Appendix D).

The intention to use antibiotics appropriately was common to both patterns A and B and was assessed using five items. We used a 6-point Likert scale ranging from 1 (strongly disagree) to 6 (strongly agree). The scale was reversed in the analysis.

**Fig. 2:**
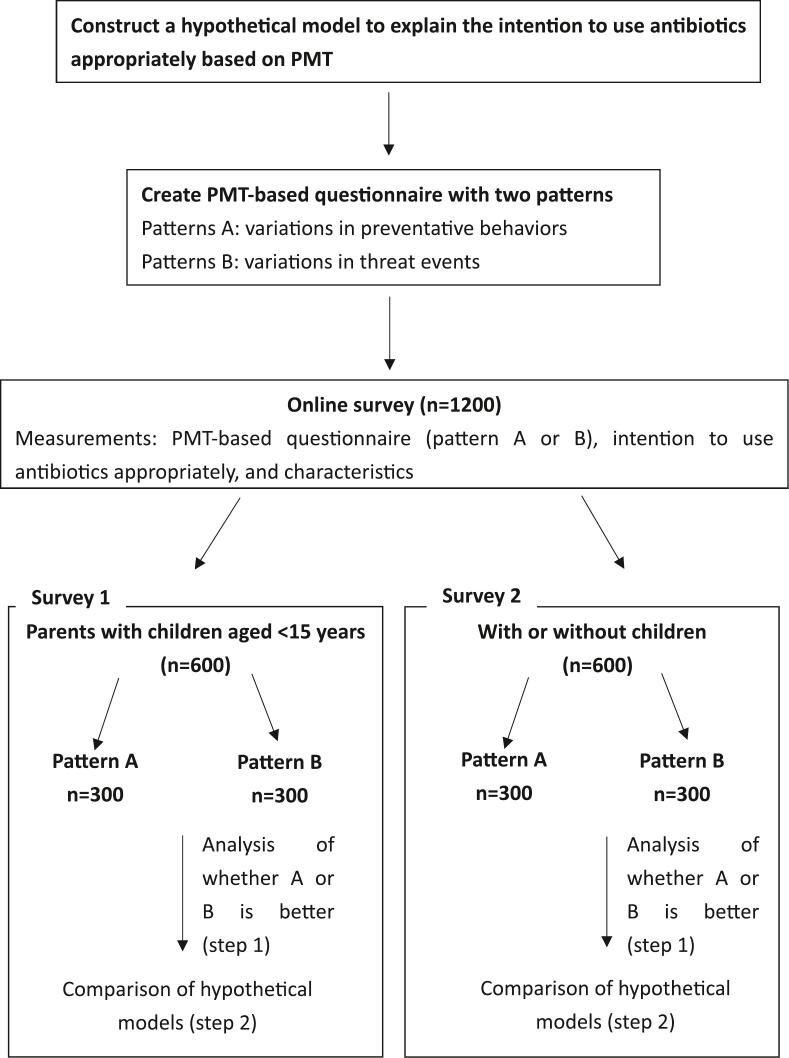
Flowchart of the survey. Figure showing the flow of the survey. Survey 1: Parental intent to use antibiotics appropriately in pediatric antibiotic use. Survey 2: Adults' intent to use antibiotics appropriately in antibiotics use.

We also collected data on various characteristics including age, sex, residence, hospitalization experience, work pattern, presence of a child, age of the youngest child, history of antibiotic use in the child, history of hospitalization, and presence of caregivers when the child was sick. Fig. 2 shows a flowchart of the survey.

### 2.2 Participants

Participants were recruited through Cross Marketing, Inc. The inclusion criteria were as follows: 1) individuals aged 20–69 years and 2) individuals who had ever taken antibiotics. The exclusion criteria were individuals and family members of healthcare workers. The participants received rewards based on the survey company's regulations.

Survey 1 participants included parents with children aged <15 years. Patterns A and B had 300 participants each with a sex ratio of 1:1.

Survey 2 included 300 participants for patterns A and B each. For each pattern, 60 participants in their 20s–60s were recruited with a sex ratio of 1:1.

### 2.3 Data analysis

This analysis was conducted in two steps. IBM SPSS (version 27.0; IBM Corp., Armonk, NY, USA) was used for statistical analyses and IBM SPSS AMOS (version 26.0) was used for structural equation modeling (SEM). For all analyses, *p* < 0.05 was considered statistically significant. For SEM, a minimum sample size of 200–250 was considered necessary [21,22]; thus, the sample size was set to 300 participants for each pattern. The basic attributes of the two groups, patterns A and B, were compared in each survey. In surveys 1 and 2, the χ^2^ test was used to assess the difference between patterns A and B in terms of each work pattern and hospitalization experience. In survey 1, a *t*-test was used to evaluate the age of participants and the youngest child, and the χ^2^ test was used to assess the history of antibiotic use in children, hospitalization history of the child, and presence of caregivers when the child was sick. To determine the intention to use antibiotics appropriately, Cronbach's alpha coefficients for patterns A and B in surveys 1 and 2 were calculated, and the total score was used for analysis in step 2 to identify issues.

#### 2.3.1 Step 1: validation process of PMT questionnaire

For the questionnaire items of the PMT constructs, we checked for ceiling effects that were defined as the mean + 1 SD exceeding the upper limit of possible values for the data and the floor effects that were defined as the mean − 1 SD exceeding the lower limit of possible values for the data. Cronbach's alpha was used to evaluate the internal consistency of the six PMT constructs. A Cronbach's alpha coefficient of 0.7 or higher is considered desirable [23]. Therefore, Cronbach's alpha coefficient was calculated for each construct and items to be considered for inclusion in the calculation of the total score. The questionnaire items were deleted to increase the Cronbach's alpha coefficient, and the total score was calculated. Patterns with no ceiling or floor effects, unbiased distribution, and many items with high Cronbach's alpha coefficients were used in step 2. To evaluate the validity, a six-factor confirmatory factor analysis was conducted using the pattern that was decided for use in step 2.

#### 2.3.2 Step 2: comparison of hypothetical models and assessment of the potential of the PMT constructs to explain the intent to use antibiotics appropriately

**Fig. 3:**
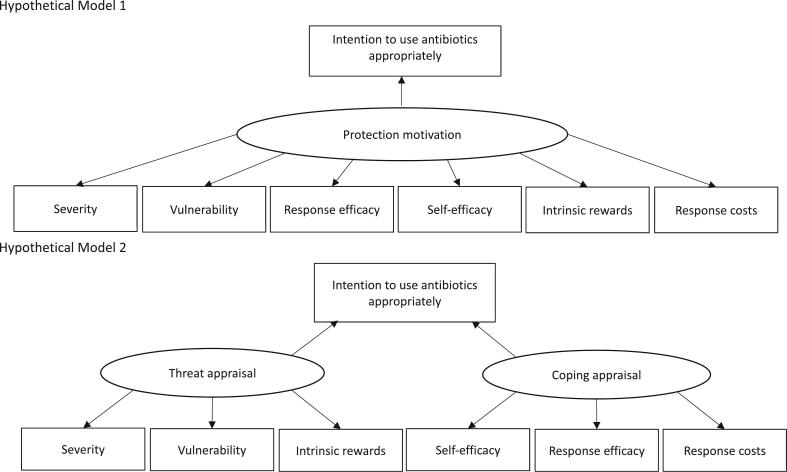
Hypothetical models. Hypothetical Model 1 proposed that protection motivation, consisting of six the protection motivation theory (PMT) constructs, influences the intention to use antibiotics appropriately. Hypothesis Model 2 proposed that two factors, threat appraisal consisting of severity, vulnerability, and intrinsic reward and coping appraisal consisting of self-efficacy, response efficacy, and response costs, influence the intent to use antibiotics appropriately.

PMT comprises two processes: threat appraisal and coping appraisal. Threat appraisal evaluates maladaptive behaviors based on severity, vulnerability, and intrinsic rewards. Severity and vulnerability inhibit maladaptive behaviors, whereas intrinsic rewards facilitate them. Coping appraisal evaluates adaptive behavior based on response efficacy, self-efficacy, and response costs. Response efficacy and self-efficacy facilitate preventive behaviors, whereas response costs inhibit them [12]. We hypothesized two models for the association between the six PMT constructs and the intention to use antibiotics appropriately (Fig. 3). Hypothetical Model 1 proposed that protection motivation, consisting of six PMT constructs, influences the intention to use antibiotics appropriately. PMT hypothesizes that protection motivation is evoked by two processes: threat appraisal and coping appraisal. Based on this hypothesis, Hypothesis Model 2 proposed that two factors, threat appraisal and coping appraisal, influence the intent to use antibiotics appropriately. SEM was used to validate the hypothesized model. Model fit was evaluated using the model χ^2^ test, comparative fit index (CFI), Tucker Lewis index (TLI), root mean square error of approximation (RMSEA), and standardized root mean square residual (SRMR). The model was defined to have a good fit if the χ^2^ value was not statistically significant, CFI and TLI values were ≥ 0.90, and RMSEA and SRMR were ≤ 0.08 [24,25].

If the hypothesized model did not fit, SEM was repeated to search for a new model.

## 3 Results

### 3.1 Descriptive statistics

Table 1 presents the descriptive data of the overall sample. No significant difference was observed in work pattern between patters A and B with respect to the pattern in either survey 1 (χ^2^ = 4.20, *p* = 0.38) or survey 2 (χ^2^ = 1.52, *p* = 0.82) and in the hospitalization experience in either survey 1 (χ^2^ = 0.12, *p* = 0.73) or survey 2 (χ^2^ = 2.06, *p* = 0.15). In survey 1, the mean participant age was 43.9 (±7.6) years for pattern A and 44.6 (±6.7) years for pattern B, with no significant difference (*p* = 0.290) between the two patterns. The mean ages of the youngest children in patterns A and B were 7.6 (±4.4) and 8.3 (±4.4) years, respectively, with no significant difference (*p* = 0.054) between the groups. The children's experience with antibiotics accounted for 228/300 (76.0%) in pattern A and 219/300 (73.0%) in pattern B. No significant difference was observed in the children's experience with antibiotics (χ^2^ = 2.06, *p* = 0.15), hospitalization (χ^2^ = 2.06, *p* = 0.15), or the presence of a caregiver for the child (χ^2^ = 2.06, *p* = 0.754) between patterns A and B.

**Table 1:** Descriptive statistics.

		Survey 1									Survey 2								
		Pattern A				Pattern B					Pattern A				Pattern B				
Variable	Category	(*n* = 300)				(*n* = 300)			χ^2^	*p*	(n = 300)				(n = 300)			χ^2^	*p*
Age: Mean (SD)		43.9 (7.6)				44.6 (6.7)				0.29^a^	44.8 (14.0)				44.9 (14.0)				
Sex: n (%)																			
	Male	150	(50.0)			150	(50.0)				150	(50.0)			150	(50.0)			
	Female	150	(50.0)			150	(50.0)				150	(50.0)			150	(50.0)			
Residence: n(%)																			
	Hokkaido	7	(2.3)			14	(4.7)				21	(7.0)			15	(5.0)			
	Tohoku	12	(4.0)			20	(6.7)				11	(3.7)			12	(4.0)			
	Kanto	120	(40.0)			117	(39.0)				128	(42.7)			107	(35.7)			
	Chubu	44	(14.7)			55	(18.3)				47	(15.7)			52	(17.3)			
	Kinki	62	(20.7)			48	(16.0)				53	(17.7)			68	(22.7)			
	Chugoku	21	(7.0)			17	(5.7)				15	(5.0)			14	(4.7)			
	Shikoku	13	(4.3)			5	(1.7)				8	(2.7)			6	(2.0)			
	Kyushu	21	(7.0)			24	(8.0)				17	(5.7)			26	(8.7)			
Work pattern: n(%)																			
	Regular employee	150	(50.0)			164	(54.7)		4.20	0.38^b^	115	(38.3)			120	(40.0)		1.52	0.82^b^
	Independent business	23	(7.7)			18	(6.0)				17	(5.7)			23	(7.7)			
	Part-time or temporary worker	54	(18.0)			58	(19.3)				65	(21.7)			63	(21.0)			
	Full-time Homemaker	68	(22.7)			52	(17.3)				55	(18.3)			52	(17.3)			
	Other	5	(1.3)			8	(2.7)				48	(16.0)			42	(14.0)			
Experienced hospitalization: n(%)		198	(66.0)			194	(64.7)		0.12	0.73^b^	197	(65.7)			180	(60.0)		2.06	0.15^b^
Age of their youngest child: Mean (SD)		7.6 (4.4)				8.3 (4.4)				0.05^a^									
Has experience of children receiving antibiotics: n(%)		228	(76.0)			219	(73.0)		0.71	0.40^b^									
Has experience of children being hospitalized: n(%)		123	(41.0)			122	(40.7)		0.01	0.93^b^									
Child's caregivers present: n(%)		245	(81.7)			242	(80.7)		0.10	0.75^b^									
Intention to use antibiotics appropriately: Mean (SD)																			
	**Q1–1** Self-interruption of antibiotics	4.91 (1.38)		0.79^c^		4.92 (1.38)		0.81^c^			4.38 (1.54)		0.81^c^		4.44 (1.61)		0.83^c^		
	**Q1–2** Desire to prescribe antibiotics for upper respiratory tract symptoms	4.61 (1.39)				4.74 (1.28)					4.68 (1.32)				4.68 (1.32)				
	**Q1–3** Use antibiotics left at home for upper respiratory tract symptoms,	5.02 (1.33)				5.16 (1.20)					4.84 (1.44)				4.85 (1.38)				
	**Q1–4** Desire to prescribe antibiotics for diarrhea	4.88 (1.32)				4.98 (1.23)					4.90 (1.31)				4.83 (1.31)				
	**Q1–5** Use antibiotics left at home for diarrhea	5.18 (1.20)				5.26 (1.09)					4.98 (1.32)				4.98 (1.31)				

SD: Standard deviation.

Survey 1: Parental intent to use antibiotics appropriately in pediatric antibiotic use.

Survey 2: Adults' intent to use antibiotics appropriately in antibiotics use.

a *t*-test

b χ^2^ test.

c Cronbach's alpha coefficient.

The Cronbach's alpha coefficient for the five items of the intention to use antibiotics appropriately was >0.7 in both surveys. Therefore, the sum of the five items was used to represent the intention to use antibiotics appropriately.

### 3.2 Step 1: validation process of PMT questionnaire

The response distribution and Cronbach's alpha coefficients for the PMT constructs' questionnaire items are presented in Table 2. Regarding the severity in survey 1 of pattern A, the Cronbach's alpha coefficient including “Diseases caused by antimicrobial resistance is a problem only for me” was 0.71, which increased to 0.91 after excluding this item. Similarly, the Cronbach's alpha coefficient for severity in survey 2, which included the same item, was 0.60, but increased to 0.82 after excluding it. Therefore, the item “Diseases caused by antimicrobial resistance is a problem only for me” was excluded in both surveys. In survey 2 of pattern A, response efficacy did not have good internal consistency with α < 0.7, whereas pattern B had higher Cronbach's alpha coefficients for all PMT constructs. In addition, to compare the distribution of responses among the patterns, we ranked the questionnaire items with the mean value of the distribution of responses for each question item close to “4. neutral,” which was the center of the scale for each of the PMT constructs, patterns A and B, for each survey. When the top three questionnaire items were assessed for each PMT construct in both surveys 1 and 2, pattern A had 5/18 items (27.8%) and pattern B had 13/19 items (68.4%). Therefore, pattern B was adopted for step 2.

**Table 2:** Response distribution of protection motivation theory constructs.

	Pattern A						Pattern B					
	Item	Survey 1			Survey 2		Item	Survey 1			Survey 2	
Constructs		Mean (SD)	α		Mean (SD)	α		Mean (SD)	α		Mean (SD)	α
Severity	Diseases caused by antimicrobial resistance is a serious problem for you.	4.44 (1.64)	0.91		4.22 (1.67)	0.82	Antimicrobial resistance can make disease symptoms more severe.	4.24 (1.52)	0.93		4.68 (1.46)	0.92
	Diseases caused by antimicrobial resistance is a serious social problem.	4.61 (1.63)			4.84 (1.49)		Antimicrobial resistance may prevent successful treatment of various infections.	4.49 (1.55)			4.89 (1.41)	
	Diseases caused by antimicrobial resistance are not just somebody else's problem.	4.89 (1.61)			4.98 (1.48)		Antimicrobial resistance may reduce the number of antibiotics that can be used in the future.	4.51 (1.61)			4.76 (1.49)	
Vulnerability	Uncontrolled use of antibiotics increases the likelihood of disease caused by antimicrobial resistance.	4.41 (1.47)	0.88		4.76 (1.47)	0.88	The use of antibiotics when they are unnecessary increases the likelihood that a variety of infections will not be successfully treated.	4.49 (1.43)	0.94		4.70 (1.37)	0.93
	Easy use of antibiotics increases the likelihood of diseases caused by antimicrobial resistance.	4.30 (1.52)			4.63 (1.49)		The use of antibiotics when they are unnecessary increases the likelihood that the disease will become more severe.	4.26 (1.39)			4.51 (1.34)	
	Using antibiotics contrary to a doctor's instructions increases the likelihood of diseases caused by antimicrobial resistance.	4.48 (1.50)			4.64 (1.44)		The use of antimicrobials when they are unnecessary increases the likelihood that fewer antibiotics will be available in the future.	4.55 (1.52)			4.70 (1.48)	
Response efficacy	By not requesting antibiotics when not prescribed, diseases caused by antimicrobial resistance can be avoided.	4.12 (1.37)	0.79		4.36 (1.36)	0.68	The treatment of various infections can be accomplished by not demanding more antibiotics when they are not prescribed.	4.08 (1.26)	0.92		4.21 (1.26)	0.92
	By not using excess antibiotics at home at one's discretion, diseases caused by antimicrobial resistance can be avoided.	4.17 (1.52)			4.24 (1.53)		Prevent the severity of the illness by not demanding more antibiotics when they are not prescribed.	4.01 (1.21)			4.16 (1.25)	
	By taking all prescribed antibiotics without stopping at one's discretion, diseases caused by antimicrobial resistance can be avoided.	4.63 (1.46)			4.69 (1.50)		More antibiotics are available in the future by not demanding antibiotics when they are not prescribed.	4.23 (1.40)			4.46 (1.26)	
Self-efficacy	You can consider yourself fine without antibiotics if they are not prescribed.	4.99 (1.46)	0.81		5.18 (1.45)	0.73	You can avoid a visit to another hospital to get a prescription for antibiotics if you were not prescribed them.	4.72 (1.68)	0.85		5.13 (1.41)	0.85
	You can avoid using extra antibiotics at home at your discretion.	5.14 (1.64)			5.07 (1.61)		You can choose not to tell your doctor that you want him or her to prescribe antibiotics if you were not prescribed them.	4.97 (1.59)			4.98 (1.50)	
	You can take your prescribed antibiotic all the way through without stopping at your discretion.	5.22 (1.51)			5.19 (1.50)		You can choose not to use your extra antibiotics if you were not prescribed them.	5.05 (1.69)			5.04 (1.51)	
Intrinsic rewards	You feel more comfortable using an antibiotic even if your doctor says they are not necessary.	2.96 (1.50)	0.91		2.97 (1.44)	0.91	You feel more comfortable using an antibiotic even if your doctor says they are not necessary.	3.05 (1.54)	0.94		3.19 (1.58)	0.93
	You are more satisfied with the use of antibiotics even if your doctor says they are unnecessary.	2.96 (1.53)			2.93 (1.48)		You are more satisfied with the use of antibiotics even if your doctor says they are unnecessary.	2.98 (1.52)			3.03 (1.51)	
	You trust antibiotics more than fever reducers, cough medicines, etc., even if your doctor says they are unnecessary.	2.92 (1.47)			2.94 (1.46)		You trust antibiotics more than fever reducers, cough medicines, etc., even if your doctor says they are unnecessary.	2.98 (1.47)			3.02 (1.49)	
Response costs	If antibiotics were not prescribed, it would be to your detriment not to use extra antibiotics at home at your discretion.	2.80 (1.54)	0.86		2.73 (1.51)	0.87	You are concerned that you will become seriously ill by not using an antibiotic, even if your doctor tells you they are unnecessary.	3.09 (1.49)	0.95		3.19 (1.46)	0.95
	If you were not prescribed an antibiotic, it would be to your detriment not to request more.	2.56 (1.33)			2.57 (1.39)		You are concerned that you will prolong your illness by not using an antibiotic, even if your doctor tells you they are unnecessary.	3.19 (1.55)			3.22 (1.49)	
	It would be to your detriment to take the prescribed antibiotics without stopping at your discretion.	2.65 (1.49)			2.56 (1.44)		Even if the doctor says antibiotics are unnecessary, not using them will interfere with daily life.	2.82 (1.43)			2.94 (1.44)	
							Even if the doctor says antibiotics are unnecessary, not using them increases the probability of a return visit to the doctor.	3.03 (1.47)			3.09 (1.42)	

Survey 1: Parental intent to use antibiotics appropriately in pediatric antibiotic use.

Survey 2: Adults' intent to use antibiotics appropriately in antibiotic use.

To evaluate the validity, a six-factor confirmatory factor analysis was conducted using pattern B. In survey 1, the goodness-of-fit index was χ^2^ (df) = 495.42 (137), *p* < 0.001, CFI = 0.941, TLI = 0.927, RMSEA = 0.094, and SRMR = 0.040; the model was a good fit with the exception of RMSEA, which also passed the upper limit of <0.10. The path coefficients of the factors for each item were all statistically significant, ranging from 0.80 to 0.95. In survey2, the goodness-of-fit index was χ^2^ (df) = 382.22 (137), *p* < 0.001, CFI = 0.955, TLI = 0.944, RMSEA = 0.077, and SRMR = 0.044, indicating a good fit. The path coefficients of the factors for each item were all statistically significant and ranged from 0.81 to 0.99.

### 3.3 Step 2: analysis of hypothetical models and assessment of the potential of the PMT constructs to explain the intent to use antibiotics appropriately

#### 3.3.1 SEM of hypothetical models

The hypothetical model defined in this study did not fit (Hypothesis model 1: Survey 1 χ^2^ (df) = 678.79 (14), *p* < 0.001, CFI = 0.387, TLI = 0.080, RMSEA = 0.399, SRMR = 0.282, survey 2 χ^2^ (df) = 595.81 (14), *p* < 0.001, CFI = 0.454, TLI = 0.181, RMSEA = 0.373, SRMR = 0.256 and Hypothesis model 2: Survey 1 χ^2^ (df) = 424.39 (12), *p* < 0.001, CFI = 0.918, TLI = 0.856, RMSEA = 0.158, SRMR = 0.089, survey 2 χ^2^ (df) = 593.92 (12), *p* < 0.001, CFI = 0.918, TLI = 0.851, RMSEA = 0.159, SRMR = 0.107). Therefore, a new model was explored.

#### 3.3.2 Model reconstruction

**Fig. 4:**
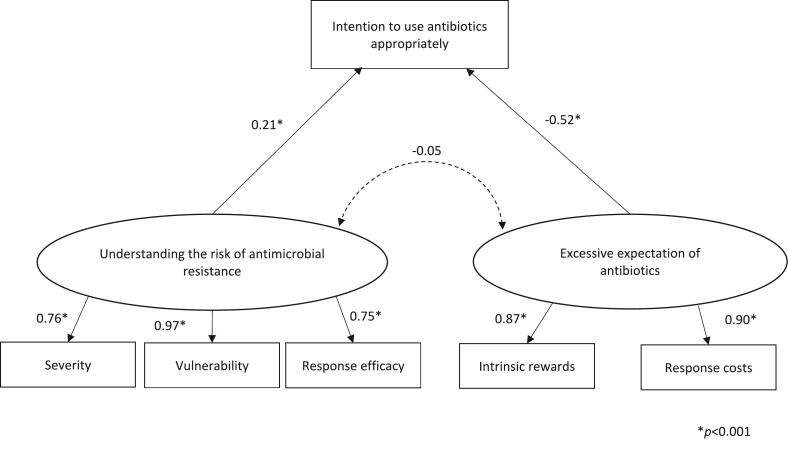
Final model of pediatric antibiotic use. This is a fit model with structural equation modeling (SEM) performed for the intention to use antibiotics appropriately and the protection motivation theory (PMT) constructs in pediatric antibiotics use. χ^2^ (df) = 12.88 (7), *p* = 0.075, comparative fit index (CFI) = 0.993, Tucker Lewis index (TLI) = 0.985, root mean square error of approximation (RMSEA) = 0.053, standardized root mean square residual (SRMR) = 0.035.

SEM was repeated with the six PMT constructs and the intention to use antibiotics appropriately in surveys 1 and 2, respectively (Table 3). In survey 1, severity, vulnerability, and response efficacy formed one factor, whereas intrinsic rewards and response costs formed another. A model was fitted in which a path was drawn from each factor toward the intention to use antibiotics appropriately (χ^2^ (df) = 12.88 (7), *p* = 0.075, CFI = 0.993, TLI = 0.985, RMSEA = 0.053, SRMR = 0.035). The factor comprising severity, vulnerability, and response efficacy was “understanding the risk of antimicrobial resistance” (“Understanding the risk”), and the factor consisting of intrinsic rewards and response costs was “Excessive expectation of antibiotics” (“Excessive expectation”). This model is presented in Fig. 4. The path coefficient from “Excessive expectation” toward the intention to use appropriately was negative at −0.52, while “Understanding the risk” was positive at 0.21 (*p* < 0.001). Among the paths from “Understanding the risk” to severity, vulnerability, and response efficacy, the path coefficient for vulnerability was the highest (Coef. = 0.97, *p* < 0.001). Among the paths from “Excessive expectation” to intrinsic reward and response costs, the path coefficient was higher for response costs (Coef. = 0.90, *p* < 0.001).

**Table 3:** Result of structural equation modeling.

	χ^2^ value (df)	*p*	CFI	TLI	RMSEA	SRMR
Pediatric antibiotic use						
Hypothesis model 1	678.79 (14)	<0.001	0.387	0.080	0.399	0.282
Hypothesis model 2	424.39 (12)	<0.001	0.619	0.334	0.339	0.201
Final model	12.88 (7)	0.075	0.993	0.985	0.053	0.035

Adult antibiotic use						
Hypothesis model 1	595.81 (14)	<0.001	0.454	0.181	0.373	0.256
Hypothesis model 2	593.92 (12)	<0.001	0.454	0.044	0.403	0.250
Final model	0.06 (1)	0.804	1.000	1.010	0.000	0.002

CFI, comparative fit index; TLI, Tucker Lewis index; RMSEA, root mean square error of approximation; SRMR, standardized root mean square residual.

**Fig. 5:**
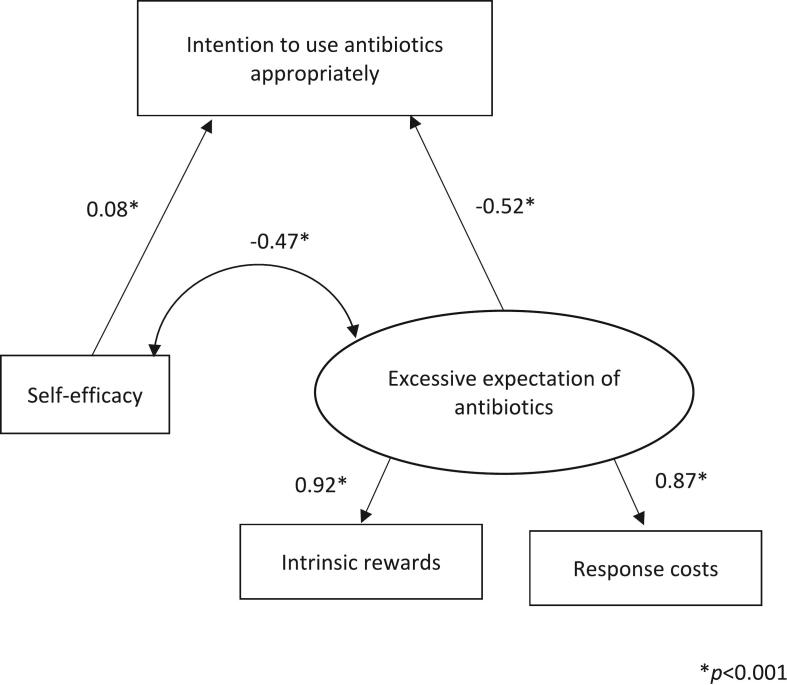
Final model of adult antibiotic use. This is a fit model with structural equation modeling (SEM) performed for the intention to use antibiotics appropriately and the protection motivation theory (PMT) constructs in adult antibiotics use. χ^2^ (df) = 0.06 (1), *p* = 0.804, comparative fit index (CFI) = 1.000, Tucker Lewis index (TLI) = 1.010, root mean square error of approximation (RMSEA) = 0.000, standardized root mean square residual (SRMR) = 0.002.

In survey 2, as in survey 1, intrinsic reward and response costs formed one factor, which was “Excessive expectation.” A model that depicted a path from “Excessive expectation” and self-efficacy to the intention to use antibiotics appropriately was fitted (χ^2^ (df) = 0.06 (1), *p* = 0.804, CFI = 1.000, TLI = 1.010, RMSEA = 0.000, SRMR = 0.002) (Fig. 5). The paths from self-efficacy and “Excessive expectation” toward the intention to use antibiotics appropriately were both significant (*p* < 0.001). The path coefficient from “Excessive expectation” toward intention to use antibiotics appropriately was negative at −0.50. The path coefficient from self-efficacy toward intention to use antibiotics appropriately was 0.11, indicating minimal impact. Among the paths from “Excessive expectation” toward intrinsic reward and response costs, the path coefficient was higher for intrinsic reward (Coef. = 0.92, *p* < 0.001). Self-efficacy was also significantly associated with “Excessive expectation” (Coef. = −0.47, *p* < 0.001).

## 4 Discussion and conclusions

### 4.1 Discussion

In this study, the hypothetical model based on PMT was not valid for pediatric or adult antibiotic use. Thus, for antibiotic use, not all six constructs based on PMT influenced the intention to use antibiotics appropriately. The structures of the models established for pediatric and adult antibiotic use varied. Thus, as hypothesized, the factors influencing the intention to use antibiotics appropriately in each population varied.

Among children, “Understanding the risk” among parents positively influenced their intention to use antibiotics appropriately, while “Excessive expectation” negatively influenced their intention. Among the PMT constructs that led to “Understanding the risk,” vulnerability had the strongest influence. The vulnerability observed in this study indicates that there is a personal risk of unnecessary antibiotic use. Previous studies have reported improvement in parental knowledge and awareness of antibiotics through various interventions [5,[26], [27], [28]]. However, these studies mainly focused on improving awareness regarding the efficacy of antibiotics, when antibiotics are needed, and behaviors that lead to antimicrobial resistance. Based on the results of this study, we suggest that in pediatric antibiotic use, the intention to use antibiotics appropriately can be improved by understanding the risks associated with the unnecessary use of antibiotics, as well as promoting awareness of the potential occurrence of these risks in children.

Furthermore, the current findings indicate that a decrease in “Excessive expectation” is a factor that improves parents' intention to use antibiotics appropriately. “Excessive expectation” was positively affected by the intrinsic rewards and response costs of PMT. These constructs indicated a sense of security and satisfaction derived from using unnecessary antibiotics, as well as anxiety and disruption in daily life caused by not using them. In a study of children with respiratory tract infections, Dalmau et al. observed no significant difference in morbidity duration when antibiotic prescriptions were postponed or not prescribed, compared to when antibiotics were prescribed immediately [29]. One way to alleviate the parents' anxiety and disruptions in daily activities is to explain to parents that even if antibiotics are deemed unnecessary at the time of consultation, changes in symptoms may necessitate their use, but the duration of illness remains the same. Educational activities to reduce excessive expectations of antibiotics could improve the intention to use antibiotics appropriately and reduce the rate of unnecessary prescriptions of antibiotics due to patients' requests.

The correlation between “Understanding the risk” and “Excessive expectation” was not significant; an improved understanding of the risk of antimicrobial resistance does not decrease excessive expectation of antibiotics. A previous study [11] reported that excessive expectations, such as misperception and strong trust in antibiotics, stemmed from mothers' past positive experiences with antibiotics. Thus, different approaches tailored to each factor are necessary to improve the intention to use antibiotics appropriately.

In adult antibiotic use, “Excessive expectation” negatively influenced the intention to use antibiotics appropriately, and self-efficacy and “Excessive expectation” were negatively correlated. As in the pediatric setting, lowering excessive expectation of antibiotics can lead to an increase in the intention to use antibiotics appropriately. Self-efficacy represents confidence in one's ability to avoid unnecessary antibiotic use. While PMT confirms that self-efficacy has a direct effect on behavioral intentions [30], these results contradict it. However, because of the suggested indirect effect of self-efficacy and intention to use antibiotics appropriately, increased self-efficacy in avoiding unnecessary antibiotics may reduce excessive expectations of antibiotics and improve the intention to use antibiotics appropriately. A study of Singaporean adults found no correlation only for antibiotic adherence and self-efficacy of PMT [31]. These results are similar to the current findings and comparable to a Japanese survey [32] on adherence to antibiotics. However, this study included the desire to have antibiotics prescribed with the intention to use antibiotics appropriately; therefore, it is possible that the indirect effect between self-efficacy and the intention to use antibiotics appropriately could be found.

The present study was conducted among parents who provide antibiotics to their children and adults who use antibiotics, and the same questionnaire items were used to investigate the intention to use antibiotics appropriately. Thus, we were able to identify differences in factors that influence the intention to use antibiotics appropriately in situations between children and adults. However, there are some limitations. One limitation of this study is that survey 1 participants included those whose children had never taken antibiotics. Therefore, they may not reflect the entire pediatric population's experience with antibiotic use. However, the results of this study are clinically important because they highlight the need to enlighten parents of children, including those who have never taken antibiotics, about the appropriate use of antibiotics. In a previous study [11], experience influenced excessive expectation of antibiotics. Therefore, the degree to which “Excessive expectation” influences intention to use antibiotics appropriately may differ when focusing only on parents of children who have consumed antibiotics in the past. In addition, the questionnaire items used in this study have not been validated in many studies and may not reflect all intentions to use antibiotics appropriately. Furthermore, the self-reported experience of using antibiotics may not reflect the actual use of antibiotics, since it is assumed that some participants may not accurately distinguish between antibiotics and other medications.

### 4.2 Innovation

This is the first study to investigate the association between PMT and the intention to use antibiotics appropriately. In addition, no study to date has investigated differences in factors that influence the intention to use antibiotics appropriately among parents who provide antibiotics to their children and adults who use antibiotics. Despite some limitations, we consider the results of this study to be important for promoting the appropriate use of antibiotics by patients.

### 4.3 Conclusion

The current findings indicate that PMT is not applicable as a framework for evaluating the factors that influence the improvement of the intention to use antibiotics appropriately in adult and pediatric patients. However, two new factors were extracted from the PMT constructs: “Excessive expectation” and “Understanding the risk.” These findings revealed that “Excessive expectation” directly influenced the intention to use antibiotics appropriately in adult antibiotics use, while among parents providing antibiotics to children, both factors directly influenced the intention to use antibiotics appropriately.

In this form, the factors influencing the intention to use antibiotics appropriately differed between adult and pediatric patients. An approach focusing on reducing excessive expectation of antibiotics would be an effective strategy for both adults and children. An approach focusing on reducing the understanding of the risk of antimicrobial resistance would be an effective strategy for children. Therefore, educational activities for appropriate antibiotic use require an approach tailored to each population's characteristics.

## Funding

This research did not receive any specific grant from funding agencies in the public, commercial, or not-for-profit sectors.

## CRediT authorship contribution statement

**Hitomi Kawamura:** Writing – review & editing, Writing – original draft, Visualization, Validation, Software, Resources, Project administration, Methodology, Investigation, Formal analysis, Data curation, Conceptualization. **Keiko Kishimoto:** Validation, Supervision, Resources, Project administration, Methodology, Funding acquisition, Formal analysis, Conceptualization.

## Declaration of competing interest

The authors declare that they have no known competing financial interests or personal relationships that could have appeared to influence the work reported in this paper.

## Figures and Tables

**Table:** 

Q1. Please answer the following questions in terms of what you think would happen to your child.							
		Strongly disagree	Disagree	Somewhat disagree	Somewhat agree	Agree	Strongly agree
1	If the symptoms go away, do you stop giving your child the prescribed antibiotics at your own discretion without finishing them? (If your child has never been prescribed antibiotics, assume they have.)	1	2	3	4	5	6
2	If you visited a health care provider because your child had a fever, sniffle, congestion, sore throat, cough, or phlegm, but the doctor told you that your child did not need antibiotics, would you ask for a prescription for antibiotics?	1	2	3	4	5	6
3	If you visited a health care provider because your child had a fever, sniffle, congestion, sore throat, cough, or phlegm, but the doctor told you that your child did not need antibiotics, would you use your own judgment in using antibiotics left at home?	1	2	3	4	5	6
4	If you visited a health care provider because your child had diarrhea, but the doctor told you that your child did not need antibiotics, would you ask for a prescription for antibiotics?	1	2	3	4	5	6
5	If you visited a health care provider because your child had diarrhea, but the doctor told you that your child did not need antibiotics, would you use your own judgment in using antibiotics left at home?	1	2	3	4	5	6


Q2. Please answer the following questions that are similar to what you think would happen to your child.								
“Antimicrobial resistance” refers to bacteria that do not respond well to antibiotics.								
		Strongly disagree	Disagree	Somewhat disagree	Neutral	Somewhat agree	Agree	Strongly agree
Severity								
1	Diseases caused by antimicrobial resistance is a serious problem for you.	1	2	3	4	5	6	7
2	Diseases caused by antimicrobial resistance is a serious social problem.	1	2	3	4	5	6	7
3	Diseases caused by antimicrobial resistance is a problem only for me.	1	2	3	4	5	6	7
4	Diseases caused by antimicrobial resistance are not just somebody else's problem.	1	2	3	4	5	6	7


Q3. Please answer the following questions as you imagine the situation in which your child will use antibiotics.								
“Antimicrobial resistance” refers to bacteria that do not respond well to antibiotics.								
		Strongly disagree	Disagree	Somewhat disagree	Neutral	Somewhat agree	Agree	Strongly agree
Vulnerability								
1	Uncontrolled use of antibiotics increases the likelihood of disease caused by antimicrobial resistance.	1	2	3	4	5	6	7
2	Easy use of antibiotics increases the likelihood of disease caused by antimicrobial resistance.	1	2	3	4	5	6	7
3	Using antibiotics contrary to a doctor's instructions increases the likelihood of disease caused by antimicrobial resistance.	1	2	3	4	5	6	7

Response efficacy								
4	By not requesting antibiotics when they are not prescribed, diseases caused by antimicrobial resistance can be avoided.	1	2	3	4	5	6	7
5	By not using excess antibiotics at home at one's discretion, diseases caused by antimicrobial resistance can be avoided.	1	2	3	4	5	6	7
6	By taking all prescribed antibiotics without stopping at one's discretion, diseases caused by antimicrobial resistance can be avoided.	1	2	3	4	5	6	7

Self-efficacy								
7	You can consider yourself fine without antibiotics if they are not prescribed.	1	2	3	4	5	6	7
8	You can avoid using extra antibiotics at home at your discretion.	1	2	3	4	5	6	7
9	You can finish your prescribed antibiotic without stopping at your discretion.	1	2	3	4	5	6	7

Intrinsic rewards								
10	You feel more comfortable using an antibiotic even if your doctor says they are not necessary.	1	2	3	4	5	6	7
11	You are more satisfied with the use of antibiotics even if your doctor says they are unnecessary.	1	2	3	4	5	6	7
12	You trust antibiotics more than fever reducers, cough medicines, etc., even if your doctor says they are unnecessary.	1	2	3	4	5	6	7

Response costs								
13	If antibiotics were not prescribed, it would be to your detriment not to use extra antibiotics at home at your discretion.	1	2	3	4	5	6	7
14	If you were not prescribed an antibiotic, it would be to your detriment not to request for it.	1	2	3	4	5	6	7
15	It would be to your detriment to take the prescribed antibiotic without stopping at your discretion.	1	2	3	4	5	6	7

**Table:** 

Q1. Please answer the following questions in terms of what you think would happen to your child.							
		Strongly disagree	Disagree	Somewhat disagree	Somewhat agree	Agree	Strongly agree
1	If the symptoms go away, do you stop giving your child the prescribed antibiotics at your own discretion without finishing them? (If your child has never been prescribed antibiotics, assume they have.)	1	2	3	4	5	6
2	If you visited a health care provider because your child had a fever, sniffle, congestion, sore throat, cough, or phlegm, but the doctor told you that your child did not need antibiotics, would you ask for a prescription for antibiotics?	1	2	3	4	5	6
3	If you visited a health care provider because your child had a fever, sniffle, congestion, sore throat, cough, or phlegm, but the doctor told you that your child did not need antibiotics, would you use your own judgment in using antibiotics left at home?	1	2	3	4	5	6
4	If you visited a health care provider because your child had diarrhea, but the doctor told you that your child did not need antibiotics, would you ask for a prescription for antibiotics?	1	2	3	4	5	6
5	If you visited a health care provider because your child had diarrhea, but the doctor told you that your child did not need antibiotics, would you use your own judgment in using antibiotics left at home?	1	2	3	4	5	6


Q2. Please answer the following questions that are similar to what you think would happen to your child.								
“Antimicrobial resistance” refers to bacteria that do not respond well to antibiotics.								
		Strongly disagree	Disagree	Somewhat disagree	Neutral	Somewhat agree	Agree	Strongly agree
Severity								
1	Antimicrobial resistance can make disease symptoms more severe.	1	2	3	4	5	6	7
2	Antimicrobial resistance may prevent successful treatment of various infections.	1	2	3	4	5	6	7
3	Antimicrobial resistance may reduce the number of antibiotics that can be used in the future.	1	2	3	4	5	6	7


Q3. Please answer the following questions as you imagine the situation in which your child will use antibiotics.								
“Antimicrobial resistance” refers to bacteria that do not respond well to antibiotics.								
		Strongly disagree	Disagree	Somewhat disagree	Neutral	Somewhat agree	Agree	Strongly agree
Vulnerability								
1	The use of antibiotics when they are unnecessary increases the likelihood that a variety of infections will not be successfully treated.	1	2	3	4	5	6	7
2	The use of antibiotics when they are unnecessary increases the likelihood that the disease will become more severe.	1	2	3	4	5	6	7
3	The use of antimicrobials when they are unnecessary increases the likelihood that fewer antibiotics will be available in the future.	1	2	3	4	5	6	7

Response efficacy								
4	The treatment of various infections can be accomplished by not demanding antibiotics when they are not prescribed.	1	2	3	4	5	6	7
5	Prevent the severity of the illness by not demanding antibiotics when they are not prescribed.	1	2	3	4	5	6	7
6	More antibiotics are available in the future by not demanding antibiotics when they are not prescribed.	1	2	3	4	5	6	7

Self-efficacy								
7	You can avoid a visit to another hospital to get a prescription for antibiotics if you were not prescribed them.	1	2	3	4	5	6	7
8	You can choose not to tell your doctor that you want him or her to prescribe antibiotics if you were not prescribed them.	1	2	3	4	5	6	7
9	You can choose not to use your extra antibiotics if you were not prescribed them.	1	2	3	4	5	6	7

Intrinsic rewards								
10	You feel more comfortable using an antibiotic even if your doctor says they are not necessary.	1	2	3	4	5	6	7
11	You are more satisfied with the use of antibiotics even if your doctor says they are unnecessary.	1	2	3	4	5	6	7
12	You trust antibiotics more than fever reducers, cough medicines, etc., even if your doctor says they are unnecessary.	1	2	3	4	5	6	7

Response costs								
13	You are concerned that you will become seriously ill by not using an antibiotic, even if your doctor tells you they are unnecessary.	1	2	3	4	5	6	7
14	You are concerned that you will prolong your illness by not using an antibiotic, even if your doctor tells you they are unnecessary.	1	2	3	4	5	6	7
15	Even if the doctor says antibiotics are unnecessary, not using them will interfere with your daily life.	1	2	3	4	5	6	7
16	Even if the doctor says antibiotics are unnecessary, not using them increases the probability of a return visit to the doctor.	1	2	3	4	5	6	7

**Table:** 

Q1. Please answer the following questions in your opinion.								
		Strongly disagree	Disagree	Somewhat disagree	Somewhat agree	Agree	Strongly agree	
1	If the symptoms go away, do you stop taking the prescribed antibiotics at your own discretion without finishing them? (If your child has never been prescribed antibiotics, assume they have.)	1	2	3	4	5	6	
2	If you visited a health care provider because you had a fever, sniffle, congestion, sore throat, cough, or phlegm, but the doctor told you that you did not need antibiotics, would you ask for a prescription for antibiotics?	1	2	3	4	5	6	
3	If you visited a health care provider because you had a fever, sniffle, congestion, sore throat, cough, or phlegm, but the doctor told you that you did not need antibiotics, would you use your own judgment in using antibiotics left at home?	1	2	3	4	5	6	
4	If you visited a health care provider because you had diarrhea, but the doctor told you that you did not need antibiotics, would you ask for a prescription for antibiotics?	1	2	3	4	5	6	
5	If you visited a health care provider because you had diarrhea, but the doctor told you that you did not need antibiotics, would you use your own judgment in using antibiotics left at home?	1	2	3	4	5	6	


Q2. Please answer the following questions as you closest match your opinion.								
“Antimicrobial resistance” refers to bacteria that do not respond well to antibiotics.								
		Strongly disagree	Disagree	Somewhat disagree	Neutral	Somewhat agree	Agree	Strongly agree
Severity								
1	Diseases caused by antimicrobial resistance are a serious problem for you.	1	2	3	4	5	6	7
2	Diseases caused by antimicrobial resistance are a serious social problem.	1	2	3	4	5	6	7
3	Diseases caused by antimicrobial resistance are a problem only for me.	1	2	3	4	5	6	7
4	Diseases caused by antimicrobial resistance are not just somebody else's problem.	1	2	3	4	5	6	7

Vulnerability								
5	Uncontrolled use of antibiotics increases the likelihood of disease caused by antimicrobial resistance.	1	2	3	4	5	6	7
6	Easy use of antibiotics increases the likelihood of disease caused by antimicrobial resistance.	1	2	3	4	5	6	7
7	Using antibiotics contrary to a doctor's instructions increases the likelihood of disease caused by antimicrobial resistance.	1	2	3	4	5	6	7

Response efficacy								
8	By not requesting antibiotics when not prescribed, diseases caused by antimicrobial resistance can be avoided.	1	2	3	4	5	6	7
9	By not using excess antibiotics at home at one's discretion, diseases caused by antimicrobial resistance can be avoided.	1	2	3	4	5	6	7
10	By taking all prescribed antibiotics without stopping at one's discretion, diseases caused by antimicrobial resistance can be avoided.	1	2	3	4	5	6	7

Self-efficacy								
11	You can consider yourself fine without antibiotics if they are not prescribed.	1	2	3	4	5	6	7
12	You can avoid using extra antibiotics at home at your discretion.	1	2	3	4	5	6	7
13	You can take your prescribed antibiotic all the way through without stopping at your discretion.	1	2	3	4	5	6	7

Intrinsic rewards								
14	You feel more comfortable using an antibiotic even if your doctor says they are not necessary.	1	2	3	4	5	6	7
15	You are more satisfied with the use of antibiotics even if your doctor says they are unnecessary.	1	2	3	4	5	6	7
16	You trust antibiotics more than fever reducers, cough medicines, etc., even if your doctor says they are unnecessary.	1	2	3	4	5	6	7

Response costs								
17	If antibiotics were not prescribed, it would be to your detriment not to use extra antibiotics at home at your discretion.	1	2	3	4	5	6	7
18	If you were not prescribed an antibiotic, it would be to your detriment not to request it.	1	2	3	4	5	6	7
19	It would be to your detriment to take the prescribed antibiotic without stopping at your discretion.	1	2	3	4	5	6	7

**Table:** 

Q1. Please answer the following questions in your opinion.								
		Strongly disagree	Disagree	Somewhat disagree	Somewhat agree	Agree	Strongly agree	
1	If the symptoms go away, do you stop taking the prescribed antibiotics at your own discretion without finishing them? (If your child has never been prescribed antibiotics, assume they have.)	1	2	3	4	5	6	
2	If you visited a healthcare provider because you had a fever, sniffle, congestion, sore throat, cough, or phlegm, but the doctor told you that you did not need antibiotics, would you ask for a prescription for antibiotics?	1	2	3	4	5	6	
3	If you visited a health care provider because you had a fever, sniffle, congestion, sore throat, cough, or phlegm, but the doctor told you that you did not need antibiotics, would you use your own judgment in using antibiotics left at home?	1	2	3	4	5	6	
4	If you visited a healthcare provider because you had diarrhea, but the doctor told you that you did not need antibiotics, would you ask for a prescription for antibiotics?	1	2	3	4	5	6	
5	If you visited a health care provider because you had diarrhea, but the doctor told you that you did not need antibiotics, would you use your own judgment in using antibiotics left at home?	1	2	3	4	5	6	


Q2. Please answer the following questions as you closest match your opinion.								
“Antimicrobial resistance” refers to bacteria that do not respond well to antibiotics.								
		Strongly disagree	Disagree	Somewhat disagree	Neutral	Somewhat agree	Agree	Strongly agree
Severity								
1	Antimicrobial resistance can make disease symptoms more severe.	1	2	3	4	5	6	7
2	Antimicrobial resistance may prevent successful treatment of various infections.	1	2	3	4	5	6	7
3	Antimicrobial resistance may reduce the number of antibiotics that can be used in the future.	1	2	3	4	5	6	7

Vulnerability								
4	The use of antibiotics when they are unnecessary increases the likelihood that various infections will not be successfully treated.	1	2	3	4	5	6	7
5	The use of antibiotics when they are unnecessary increases the likelihood that the disease will become more severe.	1	2	3	4	5	6	7
6	The use of antimicrobials when they are unnecessary increases the likelihood that fewer antibiotics will be available in the future.	1	2	3	4	5	6	7

Response efficacy								
7	The treatment of various infections can be accomplished by not demanding more antibiotics when they are not prescribed.	1	2	3	4	5	6	7
8	Prevent the severity of the illness by not demanding antibiotics when they are not prescribed.	1	2	3	4	5	6	7
9	More antibiotics are available in the future by not demanding antibiotics when they are not prescribed.	1	2	3	4	5	6	7

Self-efficacy								
10	You can avoid a visit to another hospital to get a prescription for antibiotics if you were not prescribed them.	1	2	3	4	5	6	7
11	You can choose not to tell your doctor that you want him or her to prescribe antibiotics if you were not prescribed them.	1	2	3	4	5	6	7
12	You can choose to not use your extra antibiotics if you were not prescribed them.	1	2	3	4	5	6	7

Intrinsic rewards								
13	You feel more comfortable using antibiotics, even if your doctor says they are not necessary.	1	2	3	4	5	6	7
14	You are more satisfied with the use of antibiotics even if your doctor says they are unnecessary.	1	2	3	4	5	6	7
15	You trust antibiotics more than fever reducers, cough medicines, etc., even if your doctor says they are unnecessary.	1	2	3	4	5	6	7

Response costs								
16	You are concerned that you will become seriously ill by not using an antibiotic, even if your doctor tells you they are unnecessary.	1	2	3	4	5	6	7
17	You are concerned that you will prolong your illness by not using an antibiotic, even if your doctor tells you they are unnecessary.	1	2	3	4	5	6	7
18	Even if the doctor says antibiotics are unnecessary, not using them will interfere with daily life.	1	2	3	4	5	6	7
19	Even if the doctor says antibiotics are unnecessary, not using them increases the probability of a return visit to the doctor.	1	2	3	4	5	6	7

**Table:** 

		Pattern B							
		Survey 1 (n = 300)			Survey 2 (n = 300)				
Variable	Category			adjusted residual			adjusted residual	χ^2^	*P*
Age: Mean (SD)		44.6 (6.7)			44.9 (14.0)				0.76^a^
Sex: n(%)									
	Male	150	(50.0)		150	(50.0)			
	Female	150	(50.0)		150	(50.0)			
Residence: n(%)									
	Hokkaido	14	(4.7)		15	(5.0)			
	Tohoku	20	(6.7)		12	(4.0)			
	Kanto	117	(39.0)		107	(35.7)			
	Chubu	55	(18.3)		52	(17.3)			
	Kinki	48	(16.0)		68	(22.7)			
	Chugoku	17	(5.7)		14	(4.7)			
	Shikoku	5	(1.7)		6	(2.0)			
	Kyushu	24	(8.0)		26	(8.7)			
Work pattern: n(%)									
	Regular employee	164	(54.7)	3.6	120	(40.0)	−3.6	30.75	0.00^b^
	Independent business	18	(6.0)	−0.8	23	(7.7)	0.8		
	Part-time or temporary worker	58	(19.3)	−0.5	63	(21.0)	0.5		
	Full-time homemaker	52	(17.3)	0.0	52	(17.3)	0.0		
	Other	8	(2.7)	−5.0	42	(14.0)	5.0		
Experienced hospitalization: n(%)		194	(64.7)		180	(60.0)		1.39	0.24^b^

Survey 1: Parental intent to use antibiotics appropriately in pediatric antibiotics taking situations.

Survey 2: Adults' intent to use antibiotics appropriately in their own antibiotics taking situations.

a t-test.

b χ^2^ test. SD, standard deviation.
